# Optimized production, purification, and radiolabeling of the ^203^Pb/^212^Pb theranostic pair for nuclear medicine

**DOI:** 10.1038/s41598-023-37313-8

**Published:** 2023-06-30

**Authors:** Brooke L. McNeil, Simona A. Mastroianni, Scott W. McNeil, Stefan Zeisler, Joel Kumlin, Sogol Borjian, Anthony W. McDonagh, Michael Cross, Paul Schaffer, Caterina F. Ramogida

**Affiliations:** 1 Life Sciences Division, TRIUMF, Vancouver, BC V6T 2A3 Canada; 2 Department of Chemistry, Simon Fraser University, Burnaby, BC V5A 1S6 Canada; 3 ARTMS Inc., Burnaby, BC V5A 4N5 Canada; 4 Department of Radiology, University of British Columbia, 2775 Laurel St, Vancouver, BC V5Z 1M9 Canada

**Keywords:** Analytical chemistry, Inorganic chemistry, Nuclear chemistry

## Abstract

TRIUMF is one of the only laboratories in the world able to produce both lead-203 (^203^Pb, t_1/2_ = 51.9 h) and ^212^Pb (t_1/2_ = 10.6 h) onsite via its 13 and 500 MeV cyclotrons, respectively. Together, ^203^Pb and ^212^Pb form an element-equivalent theranostic pair that potentiate image-guided, personalized cancer treatment, using ^203^Pb as a single-photon emission computed tomography (SPECT) source, and ^212^Pb for targeted alpha therapy. In this study, improvements to ^203^Pb production were accomplished by manufacturing electroplated, silver-backed thallium (Tl) targets to improve target thermal stability, which allow for higher currents during irradiation. We implemented a novel, two-column purification method that employs selective Tl precipitation (^203^Pb only) alongside extraction and anion exchange chromatography to elute high specific activity and chemical purity ^203/212^Pb in a minimal volume of dilute acid, without the need for evaporation. Optimization of the purification method translated to improvements in radiolabeling yields and apparent molar activity of lead chelators TCMC (S-2-(4-Isothiocyanatobenzyl)-1,4,7,10-tetraaza-1,4,7,10-tetra(2-carbamoylmethyl)cyclododecane) and Crypt-OH, a derivative of a [2.2.2]-cryptand.

## Introduction

Within the field of nuclear medicine, *theranostic* radiopharmaceuticals (TRPs), where *theranostic* refers to the combination of a therapeutic and diagnostic agent, enable diagnostic imaging and therapy to be conducted simultaneously, or sequentially, to allow for the development of image-guided, personalized cancer treatment plans^1^. Overall, the goal of *theranostics* is to identify the most compatible treatment option for patients to improve clinical outcome^1^. Bifunctional-chelator (BFC)-based radiopharmaceuticals for *theranostics* are composed of a radioactive metal coordinated to a bifunctional chelator attached, via a linker, to a biological targeting vector^2,3^. The vector selectively seeks out and binds to unique cell biomarkers on cancer cells to directly and selectively deliver a radioactive payload, compatible with either imaging techniques or therapy dependent on the type of radioactive decay the radiometal undergoes, to cancer cells^2,3^.

**Figure 1:**
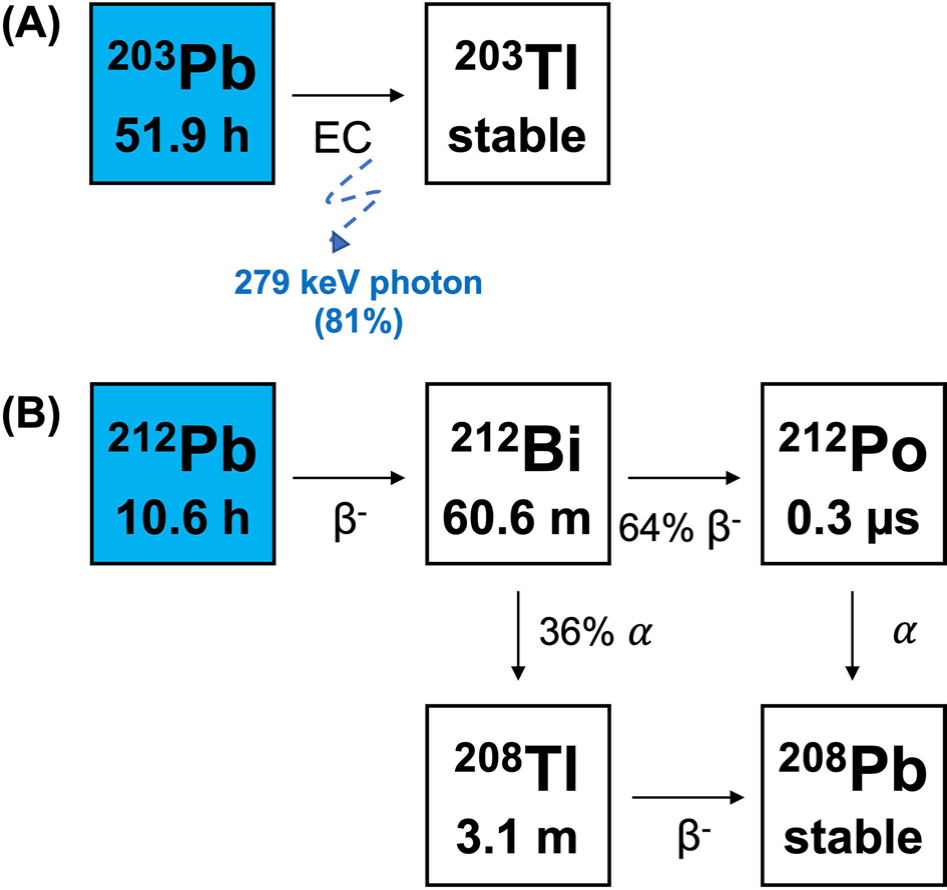
Decay scheme of (**A**) ^203^Pb and (**B**) ^212^Pb.

Recent successes in clinical trials with therapeutic isotope lead-212 (^212^Pb)-labeled radiopharmaceuticals is sparking significant interest in the potential of the element-equivalent ^203^Pb/^212^Pb theranostic pair as a means to develop image-guided, personalized cancer treatment plans for patients^4^. ^203^Pb is a diagnostic isotope that decays via electron capture, releasing a 279 keV photon (81%) compatible with single photon emission computed tomography (SPECT)^5^. ^212^Pb acts as a therapeutic isotope in this pair. Despite ^212^Pb being a pure β-emitter, it is used for targeted alpha therapy as it acts as an in vivo generator of its alpha emitting daughters ^212^Bi (t_1/2_ = 60.5 min, E_α avg_ = 6.2 MeV, 36%) and ^212^Po (t_1/2_ = 0.3 μs, E_α avg_ = 8.9 MeV, Fig. 1)^3,6^. Due to its longer half-life compared to its daughters, the use of ^212^Pb allows for increased radiopharmaceutical preparation time.

Although all components of BFC-based radiopharmaceuticals affect the success of TRPs, the importance of the specific activity of the radiometal, which refers to the amount of measured activity per unit mass of compound, is often overlooked^7^. Stable (non-radioactive) metal impurities in the radiometal solution can interfere with radiolabeling and, depending on the selectivity of the chelator, may be coordinated by the chelator. Competition with the radiometal can lower radiochemical yields (RCYs) and thus apparent molar activity (A_m_) of the radiopharmaceutical. A low A_m_ can affect kinetics and uptake at the tumour site and can lead to poor scan quality or low therapeutic effect^8^. Therefore, improving the chemical purity of the radiometal solution, and thus increasing the specific activity, is critical for advancement of TRPs. For cyclotron produced isotopes, for example ^203^Pb, the greatest chemical impurity is often the target material^9^, mandating effective separation chemistry.

In an ideal purification procedure, one should avoid the use of several columns and evaporation steps to help simplify automation and reduce risk of material loss, while utilizing a column that will remove the bulk contaminant while the isotope of interest remains bound until elution. The desired radiometal should then be eluted in a small volume of dilute acid or buffer solution to help minimize the mass of radiopharmaceutical precursor needed for labeling. In the case of cyclotron produced isotopes, it is also ideal to enable recycling of the expensive enriched target material included in the purification process to reduce costs.

^203^Pb production, particularly on a 13 MeV cyclotron like at TRIUMF, poses many challenges. ^203^Pb is produced from the proton bombardment of Tl targets. At low proton energies (i.e. 13 MeV), ^203^Pb is produced via the ^203^Tl (p,n) ^203^Pb reaction, while at higher proton energies (i.e. 24 MeV), ^203^Pb is produced via the ^205^Tl (p,3n) ^203^Pb reaction^10,11^. At 13 MeV the cross section of the ^203^Tl (p,n) ^203^Pb reaction is low at 37.4 mb^12^, limiting the amount of activity produced at the end of bombardment (EOB). Using natural Tl target, EOB yields are further limited by the lower natural abundance of ^203^Tl (29.5%) compared to ^205^Tl (70.5%)^13^. Additional complications include the low melting point of Tl (304 °C), which can cause issues with thermal stability of targets at higher currents; along with safety and contamination risks that need to be considered when using a highly toxic element such as Tl.

In our previous work on the production, purification, and radiolabeling of the ^203^Pb/^212^Pb theranostic pair, the production of ^203^Pb was limited by low thermal stability of the Tl targets, leading to a maximum allowable current of 8 μA^9^. Our previously-reported one-column purification approach, which employed extraction chromatographic PB resin, was rapid, simple, compatible with both isotopes, and eluted ^203/212^Pb in 1 M NH_4_OAc (pH 7, 3 mL), making the elute directly compatible with radiolabeling^9^. However, Tl and ^232^Th were present in the ^203^Pb and ^212^Pb elutes at concentrations of 58.2 ± 35.4 ppm and 24.3 ± 16.2 ppm, respectively. In the ^203^Pb elute, stable Pb was present at a concentration greater than 400 ppb^9^. The high concentrations of these metal impurities directly interfered with radiolabeling and contributed to the low specific activity and chemical purity that prevented, in conjunction with limitations to ^203^Pb production due to thermal instability, in vivo pre-clinical studies^9^. As a result, we focused on improving the manufacturing of the thallium target to allow for increased beam current to be applied during irradiation and on developing a novel, simple purification method that will improve the specific activity and chemical purity of the ^203/212^Pb elute that would enable (pre-)clinical use of the theranostic pair.

In this work, we describe the development of a novel target manufacturing method modified from a literature electroplating procedure^14^. These silver-backed electroplated targets have extremely high thermal stability, allowing us to produce pre-clinical amounts of ^203^Pb for imaging studies. Additionally, we have developed a novel, high-yielding purification method compatible with both ^203^Pb and ^212^Pb. This method uses selective Tl precipitation for ^203^Pb, requires minimal columns, and minimal amounts of acid. It avoids evaporation and elutes ^203/212^Pb in just 2 mL of dilute HCl. This method has also resulted in ^203/212^Pb elutes with lower concentrations of stable metal impurities compared to other methods found in literature^9,15–17^. This method is also directly compatible with Tl recycling, a rare but desirable feature that will decrease the cost of using enriched Tl targets. Optimization of the chemical purity and specific activity of ^203/212^Pb gave improved radiochemical yields (RCYs) of Pb^2+^ chelators. We anticipate this will improve the outcomes of imaging and therapy studies to come.

## Materials and methods

### Chemicals

All reagents used were purchased from commercial suppliers (Sigma Aldrich, Fisher Scientific, VWR) and were used as received, unless otherwise noted. Ultrapure hydrochloric acid (TraceSELECT), sodium hydroxide (99.99% trace metal grade), and ammonium acetate (Trace metal grade 99.99%), and Dowex-1X8 anion exchange resin (200–400 mesh, Cl form) were purchased from Fisher Scientific (Pittsburgh, PA). Ultrapure nitric acid (Environmental grade) was purchased from VWR (Radnor, PA). EDTA (99.995% trace metals basis), BRIJ-35, hydrazine hydrate (reagent grade 50–60%), thallium (I) nitrate (99.999% trace metals basis), thallium (I) sulphate (99.99% trace metals basis), 3-methyl-2-benzothiazolinonehydrazone hydrochloride (MBTH), N-(1-naphthyl)-ethylenediaminedihydrochloride (NEDA), orthophosphoric acid (ACS grade), phenol, sodium bromide, and hydrogen peroxide (30% w/w) were purchased from Millipore Sigma (St. Louis, MO). Silver sheets (14 gauge, 99.999, ¼ hard) were obtained from RioGrande (Albuquerque, NM). PB resin (Di-t-butylcyclohexano 18-crown-6, 100–150 μm particle size) was obtained from Eichrom Technologies (Lisle, IL). 1 mL poly-propylene cartridges and 1/8″polyethylene frits were purchased from United Chemical Technologies (Lewistown, PA). Natural Tl (99.99% metals basis) was purchased from Alfa Aesar (Tewksbury, MA). S-2-(4-Isothiocyanatobenzyl)-1,4,7,10-tetraaza-1,4,7,10-tetra(2-carbamoylmethyl)cyclododecane (herein referred to as TCMC) was purchased from Macrocyclics (Plano, TX) and the cryptand (Herein referred to as Crypt-OH) was synthesized as previously described^18^. Silicic acid impregnated instant thin layer chromatography paper (iTLC-SA) was purchased from Agilent Technologies (Santa Clara, CA). Deionized water was prepared on site using a MilliporeDirect-Q^®^ 3UV water purification system.

### Instrumentation

All radioactivity measurements were performed using gamma ray spectroscopy on an N-type co-axial high purity germanium (HPGe) gamma spectrometer (Canberra Industries) that has been calibrated with a 20 mL ^152^Eu and ^133^Ba source. Aliquots (5–200 μL) were removed from the samples and diluted to 20 mL for measurement at a minimum distance of 5 cm above the detector until the uncertainty of the peak area was below 5% with the dead time also kept below 5%. Analysis was performed with the Genie 2000 software package (Canberra Industries) using the 279 keV and 401 keV gamma lines for ^203^Pb measurement, and the 238 keV and 300 keV gamma lines for ^212^Pb measurement. Non-radioactive impurities in the ^203^Pb and ^212^Pb elutes were quantified using inductively coupled plasma mass spectrometry (ICP-MS) using an 8900 ICP-MS Triple Quad with a SPS 4 autosampler (Agilent Technologies, Santa Clara, CA) calibrated with calibration standards (10 ppt to 1 ppm) prepared from multi-element calibration standard 1 and 2A (Agilent Technologies, Santa Clara, CA). RadioTLC was performed using an AR-2000 TLC Scanner (Eckert and Ziegler, Valencia, CA) and radiochemical yields quantified using WinScan V3 software (Academic Software Inc.). The electroplating apparatus was obtained from ARTMS Inc. (Burnaby, BC). The set-up of the plating cell used has been previously described^19^. Spectrophotometric studies were performed using a Cary 100 UV–Visible spectrophotometer. For electroplating, the power supply used was BK Precision 9174B (B&K Precision, Yorba Linda, CA).

### Thallium targetry and cyclotron irradiation

The target disc was manufactured from a sheet of fine silver with a diameter of 28 mm and thickness of 1.5 mm and included a recess diameter of 10 mm and depth of 0.55 mm. The silver discs were polished using diamond paste (Ted Pella, Redding, CA) until a mirror finished was achieved and cleaned with water and acetone prior to plating.

The constant current electrolysis plating method used to produce the silver backed thallium targets was based on a literature method with minor deviations^14^. The plating bath was an alkaline (pH > 12.5) EDTA (0.5 M) bath with 1% hydrazine hydrate and 0.2% BRIJ-35^14^. To prepare a 100 mL plating bath, in the first beaker, 21 g of ethylenediamine tetraacetic acid (EDTA) and 5 g of sodium hydroxide were dissolved in 90 mL of deionized water and stirred until completely dissolved. Once dissolved, 2.53 mL of hydrazine hydrate and 250 μL of BRIJ-35 were added. To a second beaker, 8.475 g of natural Tl_2_SO_4_ or 8.949 g of TlNO_3_ was added and the contents of beaker 1 were transferred at a rate of 10 mL/min; once the transfer was complete, an additional 250 μL of hydrazine hydrate was added. To the plating chamber, approximately 6 mL of the plating solution was added, and electroplating occurred at a current density of 2.3 mA cm^−2^ in constant current mode for 24 h. The target was rinsed with deionized water, dried, weighed, and vacuum sealed until installation.

The Tl target was installed into a quick release solid target holder^20^ on TRIUMF’s TR13 (13 MeV) cyclotron^21^ where the 13 MeV protons are degraded to approximately 12.8 MeV by an aluminum foil (25 μm thick) used to separate the targetry system from the vacuum. Targets were irradiated at 20 μA for 2–4 h and after EOB, the target remained in the target holder for a minimum of 18 h to allow for the decay of co-produced ^202m^Pb (t_1/2_ = 3.6 h).

### Radiochemical separation of ^203^Pb

**Figure 2:**
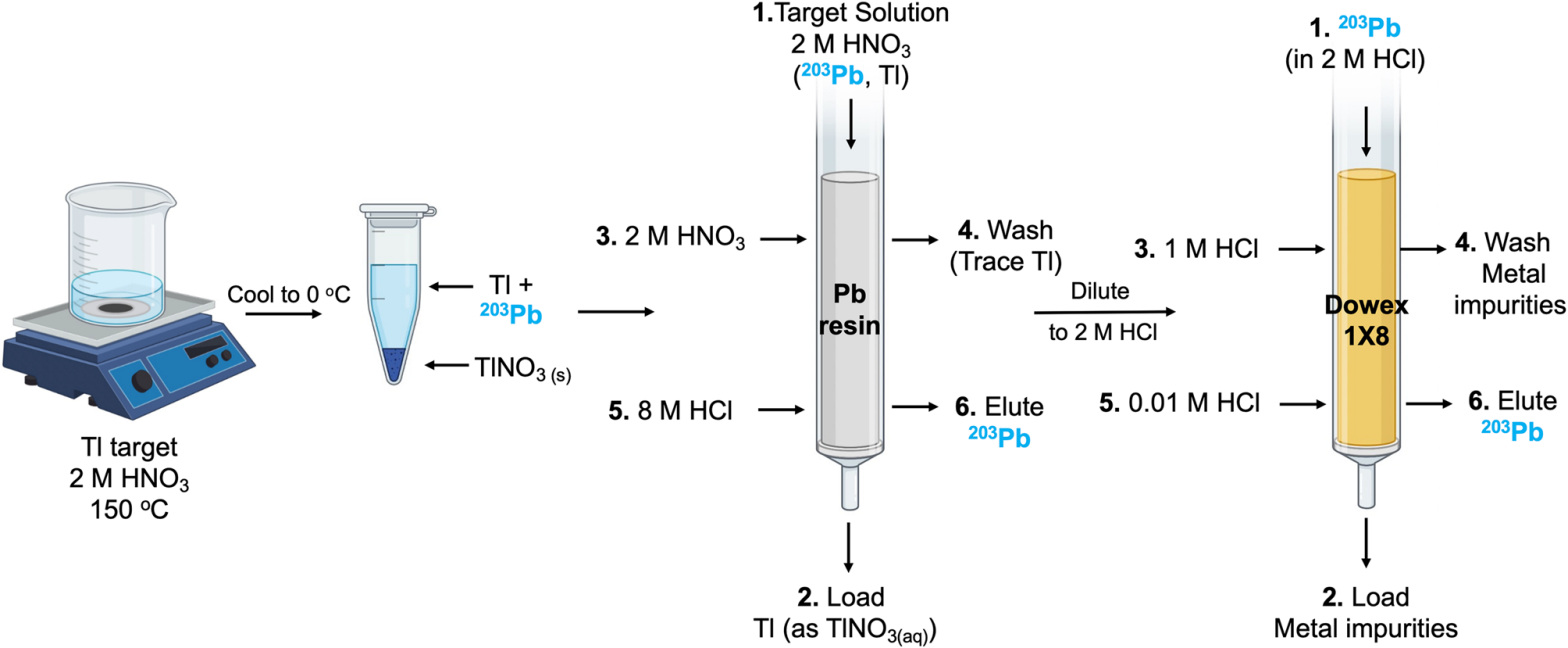
A novel ^203^Pb purification method employing selective precipitation of thallium nitrate prior to extraction via anion exchange chromatography.

Irradiated targets were dissolved in 4 mL of 2 M HNO_3_ on a hot plate at 150 °C. As soon as the dissolution of the thallium was complete, the solution was removed to reduce the dissolution of the silver target backing. The solution was then placed in a beaker of ice to precipitate Tl as TlNO_3_ by taking advantage of the difference in the solubility of TlNO_3_ in water at 100 °C (414 g/100 mL)^22^, 25 °C (9.55 g/100 mL)^22^, and 0 °C (3.90 g/100 mL)^22^. A PB resin column, composed of 60 mg of PB resin packed into a 1 mL polypropylene cartridge, was conditioned with 5 mL of deionized water followed by 5 mL of 2 M HNO_3_. The supernatant was loaded onto the PB resin column by gravity and washed with 10 mL of 2 M HNO_3_ at a flow rate of 1 mL/min to remove residual Tl and other metal impurities. The ^203^Pb was eluted from the column with 2 mL of 8 M HCl into 4, 0.5 mL fractions at a flow rate of 1.5 mL/min. The first 1 mL of eluate was diluted to 2 M HCl with 3 mL of deionized water before loading onto a second column. The diluted elute was then loaded onto a second column composed of 500 mg of Dowex-1X8 anion exchange resin (200–400 mesh, Cl form), preconditioned with 10 mL of MilliQ water followed by 10 mL of 2 M HCl. The column was washed with 0.5 mL of 1 M HCl by gravity before eluting with 0.01 M HCl at a flow rate of 1.5 mL/min into 4, 0.5 mL fractions (see Fig. 2). The yield and radionuclidic purity of the ^203^Pb in the load, wash, and elute fractions for each column were assessed via gamma spectroscopy. The chemical purity of the ^203^Pb elutes was assessed via ICP-MS to quantify the concentration of stable metal impurities.

### Recycling of Tl targets

To recycle the Tl target material, the load and wash fractions from the PB resin column of the ^203^Pb purification step were evaporated to dryness and combined with the dried thallium (I) nitrate precipitate of the thallium precipitation step. The recovered Tl (I) nitrate was then added to a plating bath solution and was used for target plating. The oxidation state and yield of the recovered thallium was determined via colorimetry^23^. Briefly, this method is based on the in-situ generation of a diazonium cation of MBTH by Tl^3+^, which reacts with NEDA to produce a blue-coloured product, whose absorbance at 590 nm is dependent on the concentration of Tl^3+^. Samples with Tl^+^ will not produce the blue product, but the Tl in these samples can still be quantified when oxidized with bromine water to oxidize the Tl^+^ to Tl^3+^.

### Preparation of ^228^Th/^212^Pb generator stock solution and radiochemical separation of ^212^Pb

**Figure 3:**
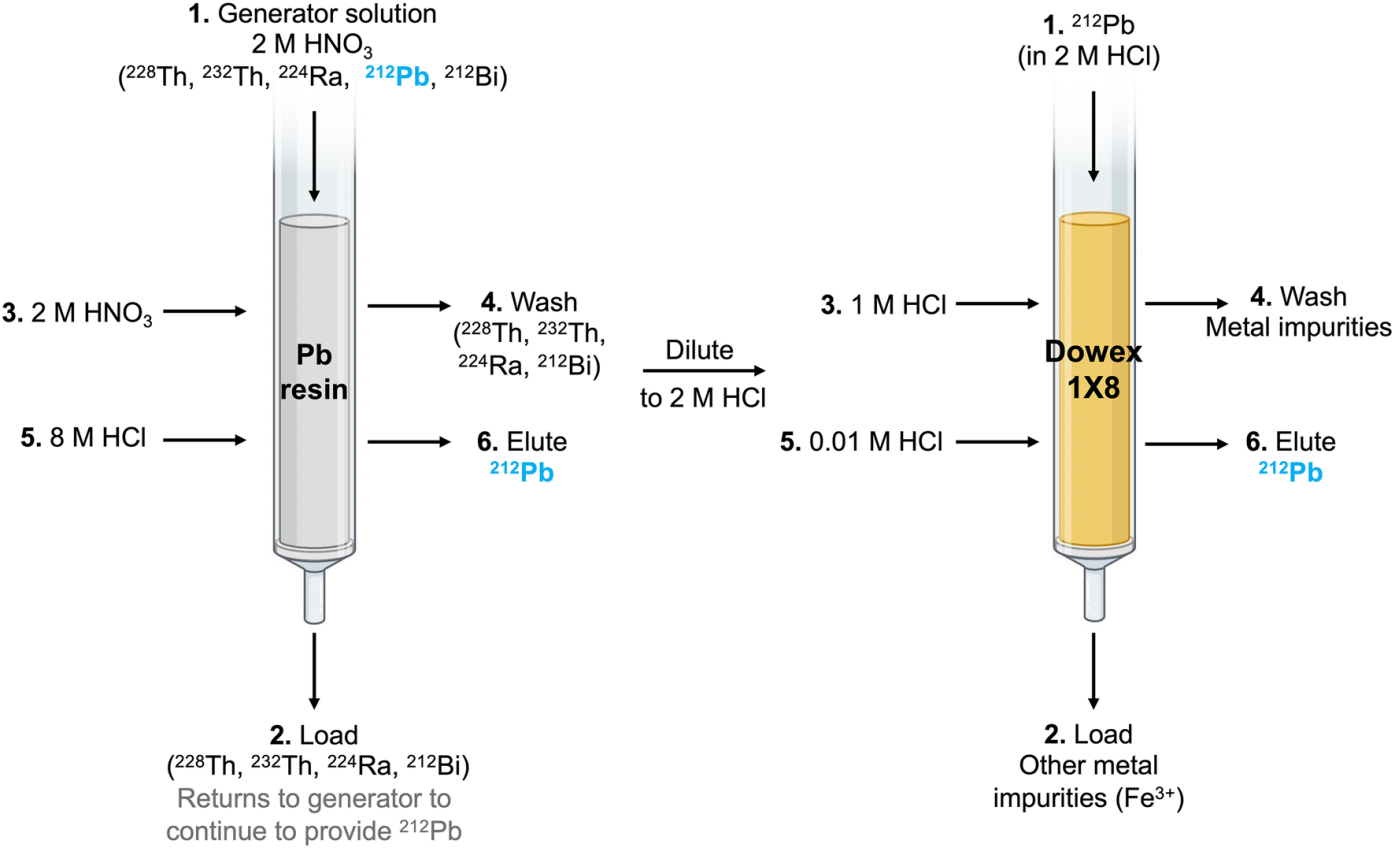
^212^Pb purification method employing extraction and anion exchange chromatography.

A ^228^Th/^212^Pb generator stock solution was prepared as previously described^9^. Briefly, thorium peroxide, precipitated out from irradiated thorium targets, was dissolved in 200 mL of 10 M HCl before loading onto a 10 mL Dowex-1X8 column^9^. The column was subsequently washed with 60 mL of 10 M HCl and then the load and wash fractions were collected and evaporated to dryness and exchanged three times with 10 M HNO_3_ before redissolving in 30 mL of 2 M HNO_3_^9^. The radiochemical separation of ^212^Pb from the ^228^Th/^212^Pb generator and the characterization of the elute product was identical to that of ^203^Pb with minor exceptions: (1) The exclusion of the thallium precipitation step and (2) that the generator stock solution was loaded onto the PB resin column at a flow rate of 1 mL/min instead of by gravity (see Fig. 3).

### Radiolabeling

Chelators TCMC and Crypt-OH were dissolved in deionized water to give stock solutions (10^–2^ M), from which serial dilutions were made to prepare chelator solutions at concentrations from 10^–3^ to 10^–8^ M. The radiolabeling reactions consisted of 10 μL of the respective chelator solution, 10 μL of 1 M NH_4_OAc (pH 7), 2 μL (for ^203^Pb, 85 kBq) to 5 μL (for ^212^Pb, 100 kBq) of the elute solution, and 78–75 μL of deionized water for a final reaction volume of 100 μL. These differences in required activity volume reflect the lower kBq/μL concentration of ^212^Pb over ^203^Pb. These reactions were all performed at ambient temperature, or 80 °C, in triplicate. Radiochemical conversion was established using iTLC-SA plates developed using EDTA (50 mM, pH 5.0). Under these conditions, the Pb^2+^ complexes remain at the baseline (R_f_ = 0) and “free”/non-complexed Pb^2+^ migrates with the solvent line (R_f_ ≅ 1). At 60 min, 10 μL aliquots were removed from each reaction mixture and analyzed. To allow for the decay of ^212^Bi (t_1/2_ = 60.6 min), plates containing ^212^Pb were measured a minimum of 10 h after development. ^203^Pb plates were measured immediately.

### ICP-MS analysis

For determination of the concentration of the stable metal impurities that may interfere with radiolabeling in the ^212^Pb and ^203^Pb elutes after the PB resin and Dowex-1X8 columns, 50 μL and 2 mL aliquots were removed, respectively, and diluted to 10 mL using 2% w/w HNO_3_.

## Results

### Targetry and cyclotron irradiation

347.6 ± 11.7 mg (n = 7) of Tl was plated onto the silver backings after 24 h at a current density of 2.3 mA cm^−2^. In an attempt to demonstrate the utility of recycled TlNO_3_, 346.1 ± 8.7 mg (n = 4) of Tl was plated under identical conditions. The deposits were occlusion- and dendrite-free mitigating the need for mechanical pressing post plating. Prior to irradiation with higher currents, test irradiations were first performed with ^nat^Tl targets using beam currents between 5 and 20 μA, for 2 to 4 h to investigate thermal stability of the target. No signs of melting were observed over this range.

### ^203^Pb isotope production and radiochemical separation

**Figure 4:**
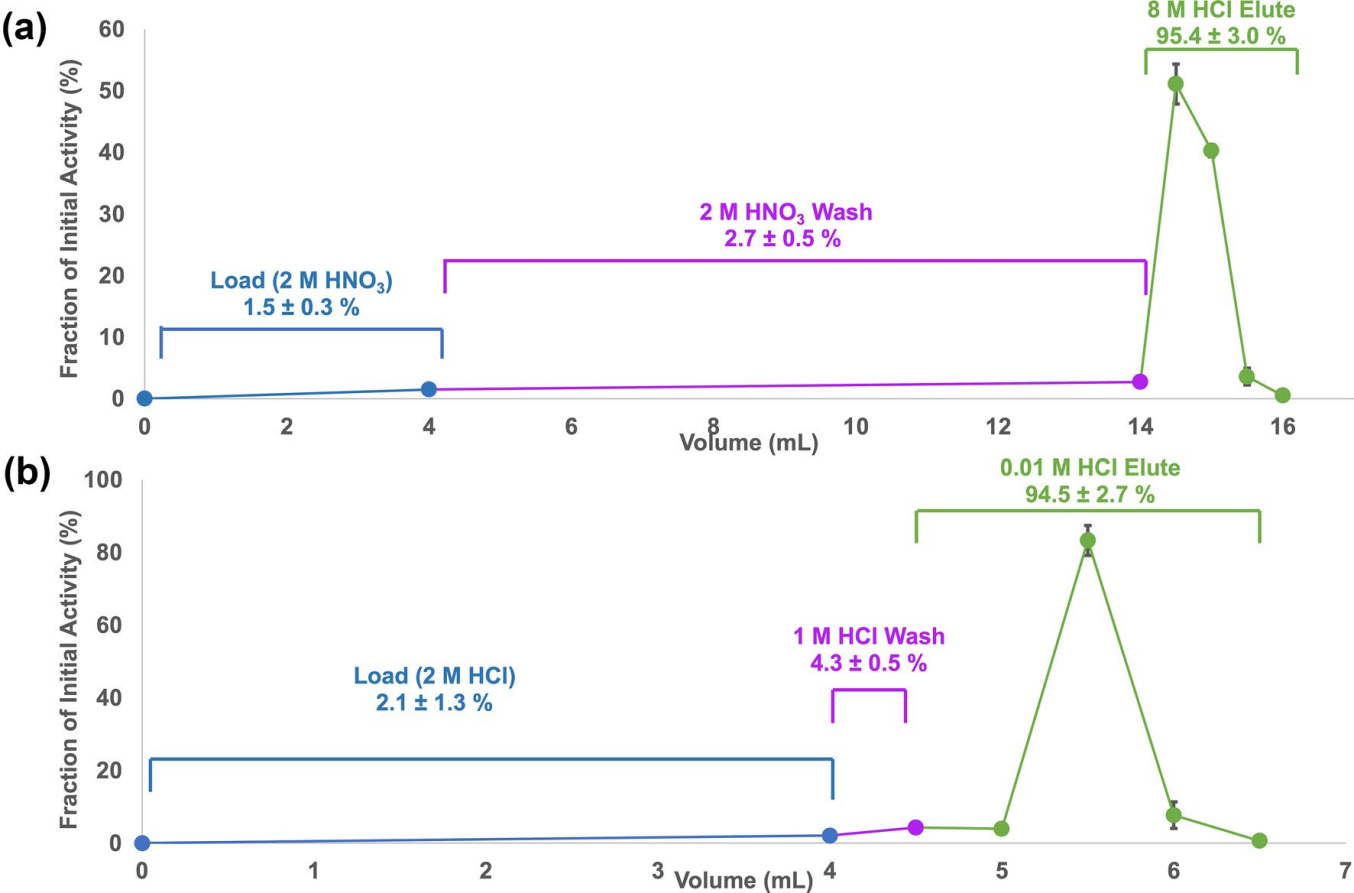
Representative elution profile of ^203^Pb purification on (**a**) PB resin (extraction chromatography) and (**b**) Dowex-1X8 resin (anion exchange chromatography) (n = 4).

On average (n = 4), 4-h 12.8 MeV proton irradiations of ^nat^Tl targets at 20 μA produced 131.8 ± 4.6 MBq of ^203^Pb at the EOB. Under the same conditions, recycled ^nat^Tl targets produced 138.7 ± 5.1 MBq (n = 3), as determined by gamma spectroscopy. The thallium metal of the target was completely dissolved after approximately 1 min, and after cooling to 0 °C, 81.0 ± 4.5% (n = 4) of the Tl metal precipitated out as solid thallium (I) nitrate; with negligible ^203^Pb in the precipitate. The load (0–4 mL) and wash (4–14 mL) fractions of the PB resin column contained 1.5 ± 0.3% and 2.7 ± 0.5% of the initial ^203^Pb activity (n = 4), respectively. These fractions were evaporated to dryness and in combination with the mass of the TlNO_3_ precipitate, 94.8 ± 1.0% (n = 4) of the initial Tl was recovered as TlNO_3_ to be reused in the plating baths for target manufacturing. With the first PB resin column, 95.4 ± 3.0% (n = 4) of the initial ^203^Pb activity was recovered in the elute with 2 mL of 8 M HCl (14–16 mL). The elution profile of ^203^Pb on the PB resin column is shown in Fig. 4a. The first 1 mL of 8 M HCl (Vol 14-15 mL), which contained 90.0 ± 2.4% (n = 4) of the initial ^203^Pb, was collected and diluted to 2 M HCl and loaded onto the Dowex-1X8 anion exchange resin where 94.8 ± 2.7% (n = 4) was eluted in 4 × 0.5 mL of 0.01 M HCl (Vol 4.5–6.5 mL), with minimal losses in the load (2.1 ± 1.3%, vol 0–4 mL) and wash (4.3 ± 0.5%, vol 4–4.5 mL), as shown in Fig. 4b. 83.3 ± 4.1% (n = 4) of the initial activity was found in the second 0.5 mL fraction (Vol 5.5 mL).

The ^203^Pb was determined to be radionuclidically pure via gamma spectroscopy (Fig. S1). The chemical purity was assessed via ICP-MS, as shown in Table 1, with the concentrations (ppb) and masses (ng) of metal impurities in the Dowex-1X8 elute. Select metal concentrations and masses from the PB resin elute in parentheses, obtained via this method are compared to values from our previously reported one-column method^9^.

**Table 1:** Metal content of ^203^Pb elute fractions in ppb (μg/L) and ng as determined by ICP-MS (n = 3).

Metal	Al	Ag	Ca	Fe	Cu	Zn	Pb	Tl
ppb (μg/L)								
One-column method^9^	168 ± 152	N.S.	568 ± 263	18 ± 11	2.7 ± 1.8	21 ± 4	495 ± 218	58,220 ± 35,392
This work	100 ± 51	1.4 ± 0.3 (In 8 M HCl elute: 3353 ± 287)	N.S.	29 ± 9	N.S.	14 ± 4	34 ± 6	26 ± 3 (In 8 M HCl elute: 2236 ± 483)
This work—recycled ^nat^Tl	N.S.	1.8 ± 0.2	N.S.	18 ± 1	N.S.	26 ± 5	25 ± 8	28 ± 14
ng								
One-column method^9^	504 ± 456	N.S.	1704 ± 789	54 ± 33	8.1 ± 5.4	63 ± 12	1,485 ± 654	174,660 ± 106,176
This work	200 ± 102	2.8 ± 0.6 (In 8 M HCl elute: 6706 ± 574)	N.S.	58 ± 18	N.S.	28 ± 8	68 ± 12	52 ± 6 (In 8 M HCl elute: 4472 ± 966)
This work—recycled ^nat^Tl	N.S.	3.6 ± 0.4	N.S.	36 ± 2	N.S.	52 ± 10	50 ± 16	56 ± 28

*N.S.* not significant.

### ^212^Pb radiochemical separation

**Figure 5:**
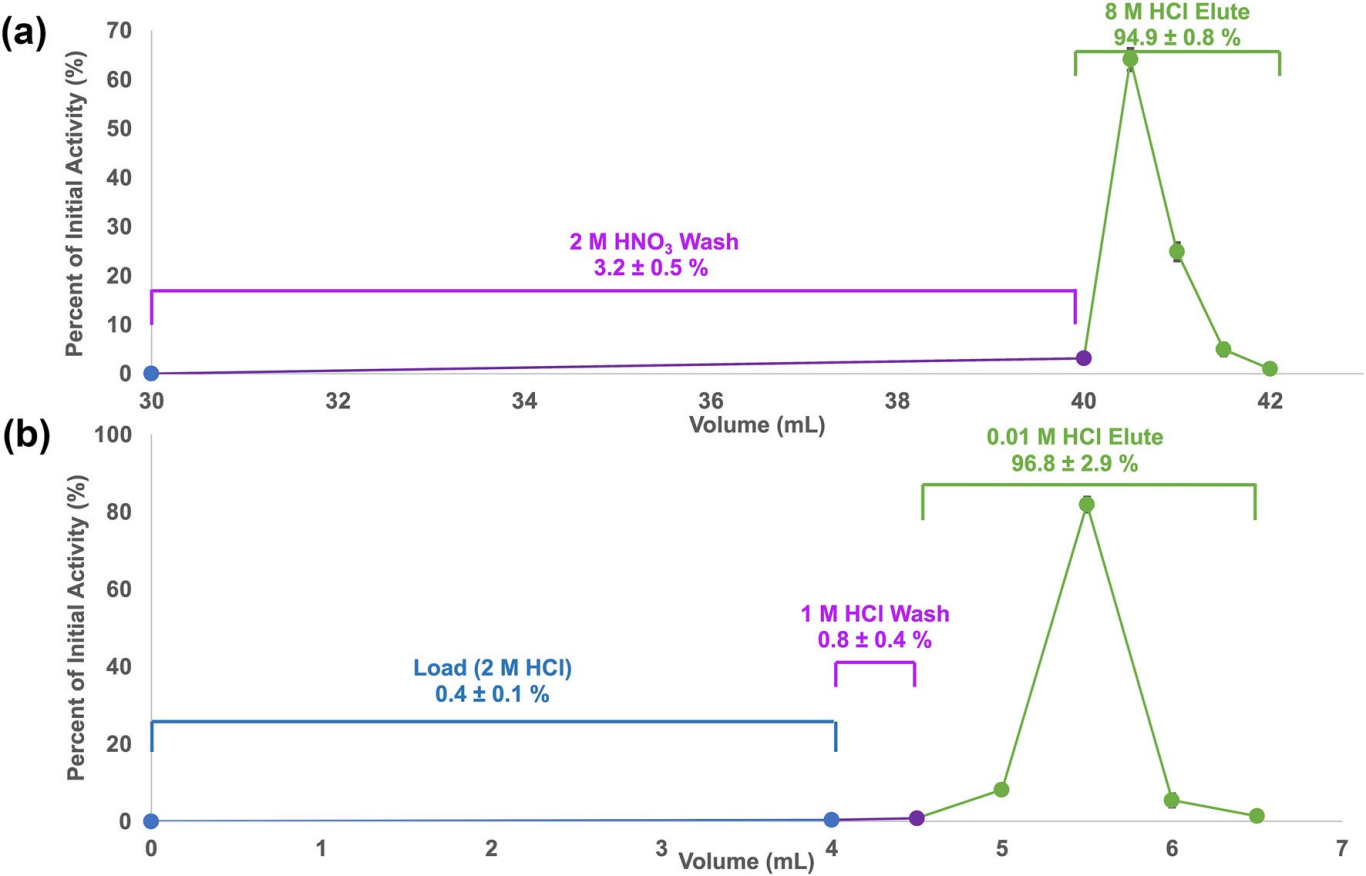
Representative elution profile of ^212^Pb purification on (**a**) PB resin (extraction chromatography) and (**b**) Dowex-1X8 resin (anion exchange chromatography) (n = 4).

The ^228^Th/^212^Pb generator stock solution, prepared from a 12,500 μA h irradiation of 8 g of ^232^Th, initially contained 22.80 ± 0.03 MBq of ^228^Th when it was processed 10 months post EOB. A 30 mL generator stock solution was first passed through the PB resin column (Fig. 3) and the load (0–30 mL) is recovered to serve as a source of ^212^Pb after equilibrium is established after approximately 2 days. Washing the PB resin with 10 mL of 2 M HNO_3_ (Vol 30–40 mL) resulted in the loss of 3.2 ± 0.5% (n = 4) of the initial ^212^Pb activity, while 94.9 ± 0.8% was eluted in 2 mL of 8 M HCl (40–42 mL, see Fig. 5a). The first 1 mL (40–41 mL) of the PB resin elute contained 89.0 ± 1.6% (n = 4) of the initial ^212^Pb activity and was loaded onto the Dowex-1X8 resin (Fig. 5b) where a minimal amount (0.4 ± 0.1%, n = 4) of the activity was lost in the load (Vol 0–4 mL), along with 0.8 ± 0.4% (n = 4) in the 1 M HCl wash (4–4.5 mL). The entire 2 mL 0.01 M HCl elute (4.5–6.5 mL) contained 96.8 ± 2.9% (n = 4) of the initial activity, with 81.9 ± 2.0% found in the second, 0.5 mL fraction (5–5.5 mL).

Similarly to ^203^Pb, ^212^Pb was determined to be radionuclidically pure (> 99.9%) via gamma spectroscopy (Fig. S2). The chemical purity was assessed via ICP-MS, as shown in Table 2, and compared to the metal concentrations and masses found in the elute of the previous one-column ^212^Pb purification method^9^.

**Table 2:** Metal contents in ^212^Pb elute fractions in ppb (μg/L) and ng as determined by ICP-MS (n = 3).

Metal	Al	Mg	Ca	Ti	Fe	Co	Ni	Cu	Zn	Pb	Th
ppb (μg/L)											
One-column method^9^	22 ± 9	612 ± 226	N.S	354 ± 168	N.S.	26 ± 11	N.S.	3 ± 2	N.S.	2 ± 2	24,352 ± 16,227
This work	2 ± 3	15 ± 7	15 ± 5	N.S.	N.S.	0.3 ± 0.2	N.S.	4 ± 2	N.S.	4 ± 2	291 ± 56 (In 8 M HCl elute: 37,650 ± 3,195)
ng											
One-column method^9^	66 ± 27	1836 ± 678	N.S.	1062 ± 504	N.S.	78 ± 33	N.S.	9 ± 6	N.S.	6 ± 6	73,056 ± 48,681 (In 8 M HCl elute: 112,950 ± 9,585)
This work	4 ± 6	30 ± 14	30 ± 10	N.S.	N.S.	0.6 ± 0.4	N.S.	8 ± 4	N.S.	8 ± 4	582 ± 112

*N.S.* not significant.

## Radiolabeling

**Figure 6:**
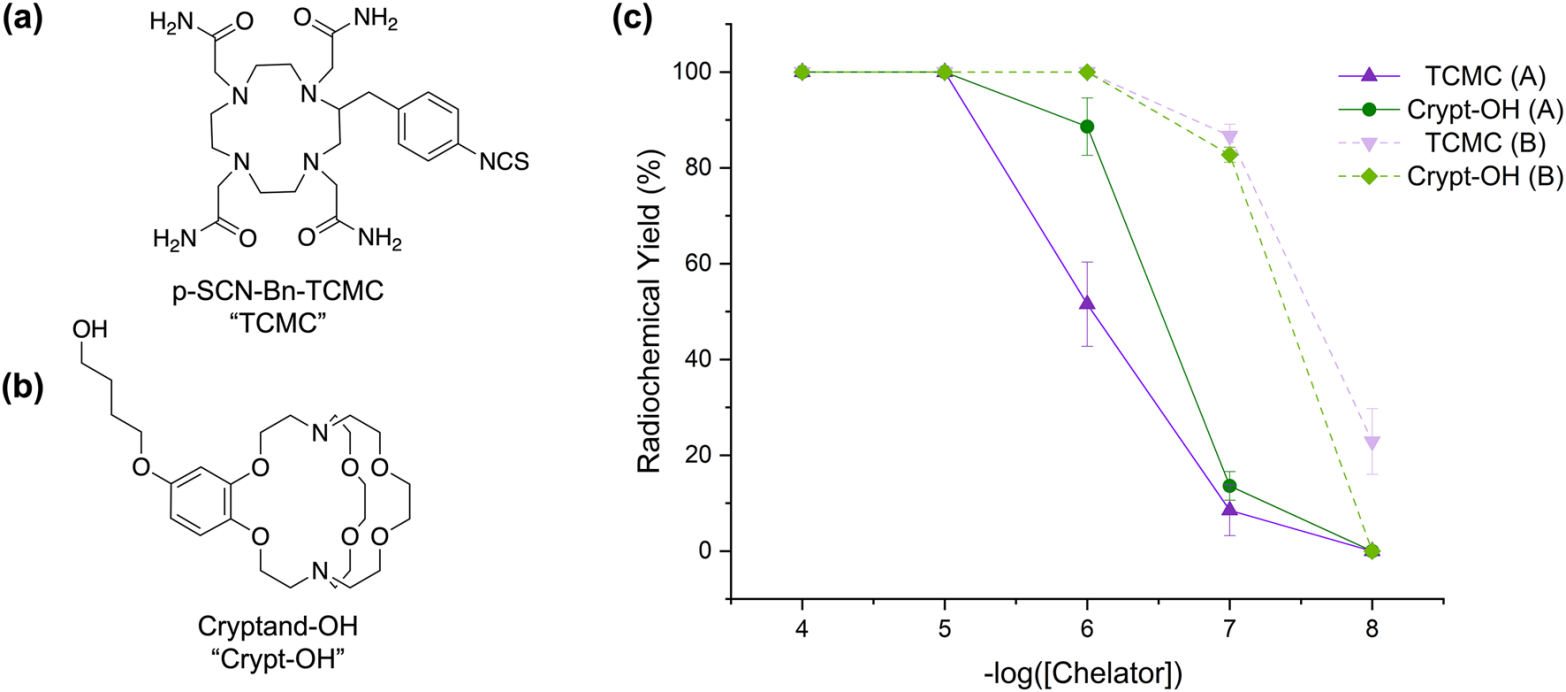
Chemical structures of (**a**) TCMC and (**b**) Crypt-OH and (**c**) radiochemical yields (%) for ^203^Pb (85 kBq) labeling reactions at pH 7 (0.1 M NH_4_OAc), room temperature, and 1 h at chelator concentrations of 10^–4^ to 10^–8^ M using ^203^Pb produced via the previous one-column (A)^9^ and new (B) method. (n = 3).

Comparative radiolabeling studies were performed to determine if there were any differences in the RCYs of chelators TCMC and Crypt-OH (see chemical structures in Fig. 6a,b, respectively) when labeled with ^203^Pb or ^212^Pb purified using the two different methods: the previous one-column procedure^9^ and the novel procedure described above. For ^203^Pb radiolabeling, the reactions were performed in triplicate at pH 7 and room temperature and the RCY (%) was determined by radio-iTLC at one hour. Using ^203^Pb isolated via the one-column method^9^, the RCYs (n = 3) of the TCMC at chelator concentrations of 10^–4^ to 10^–5^ M were quantitative and at concentrations of 10^–6^ to 10^–8^ M were 51.5 ± 8.8%, 8.5 ± 5.3%, and 0%, respectively. With the ^203^Pb isolated via the novel two-column method, quantitative RCYs were obtained at TCMC concentrations of 10^–4^ to 10^–6^ M, and were 86.7 ± 2.4% and 22.8 ± 6.9% at 10^–7^ and 10^–8^ M, respectively. By using the novel, two-column procedure, the results suggest that the A_m_ of [^203^Pb][Pb(TCMC)]^2+^ increased from 437.9 ± 38.0 MBq/μmol to 7.4 ± 0.2 GBq/μmol. For Crypt-OH, with the one-column ^203^Pb^9^, quantitative RCYs (n = 3) were achieved at concentrations of 10^–4^ and 10^–5^ M and then from 10^–6^ to 10^–8^ M, the RCYs decreased to 88.6 ± 6.0%, 13.6 ± 3.0%, and 0%, respectively. However, with the ^203^Pb obtained via the two-column method, quantitative RCYs were obtained at concentrations from 10^–4^ to 10^–6^ M and at 10^–7^ and 10^–8^ M, RCYs of 82.7 ± 1.6% and 0%, respectively, were obtained. The improvements in RCYs, as shown in Fig. 6c, suggest the A_m_ of [^203^Pb][Pb(Crypt-OH)]^2+^ increased from 753.1 ± 45.2 MBq/μmol to 7.0 ± 0.1 GBq/μmol.

With ^212^Pb, at ambient temperature RCYs for Crypt-OH were not quantitative at any concentration for either Pb-purification method. However, ^212^Pb isolated via the previously-reported one-column method saw improved RCYs (n = 3) at 80 °C between chelate concentrations of 10^–4^ to 10^–8^ M, with yields of 64.9 ± 10.7%, 36.2 ± 5.7,% 8.7 ± 1.7%, 0%, and 0%, respectively. With ^212^Pb isolated using the two-column method reported here, the RCYs increased to 100 ± 0%, 94.8 ± 4.6,% 84.3 ± 3.0,% 35.7 ± 3.8%, and 3.0 ± 0.7%, respectively. This resulted in the A_m_ of [^212^Pb][Pb(Crypt-OH)]^2+^ increasing from 13.2 ± 1.3 MBq/μmol to 1.7 ± 0.05 GBq/μmol.

**Figure 7:**
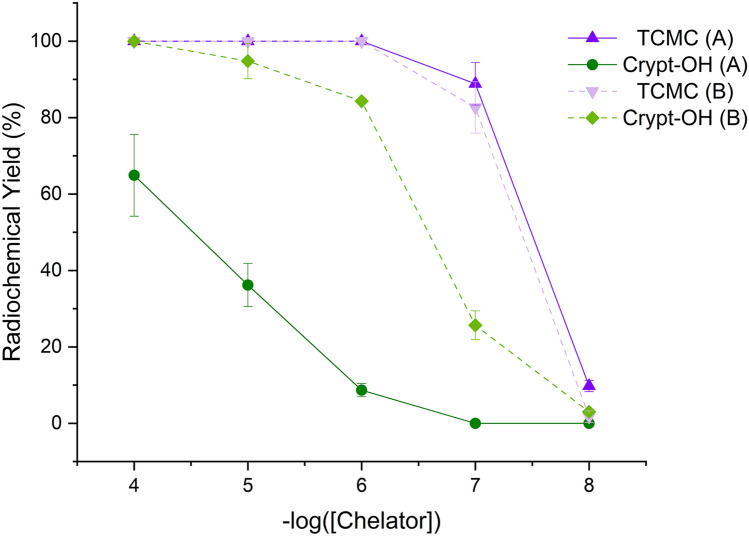
Radiochemical yields (%) for ^212^Pb (100 kBq) labeling reactions at pH 7 (0.1 M NH_4_OAc), 80 °C, and 1 h at chelator concentrations of 10^–4^ to 10^–8^ M using ^212^Pb produced via the previous one-column^9^ (A) and new (B) method. (n = 3).

With TCMC, radiolabeling was observed at both ambient temperature and 80 °C for both methods. At ambient temperature, with the one-column method, quantitative ^212^Pb RCYs were obtained for chelator concentrations of 10^–4^ and 10^–5^ M, dropping to 90.0 ± 1.6%, 48.7 ± 10.0%, and 0% between 10^–6^ to 10^–8^ M, respectively. With the two-column method, quantitative RCYs were obtained for concentrations from 10^–4^ to 10^–6^ M and yields of 50.7 ± 7.2% and 0% at chelator concentrations of 10^–7^ and 10^–8^ M, respectively, as shown in Fig. S8. At ambient temperature, the apparent molar activities of [^212^Pb][Pb(TCMC)]^2+^ were 9.7 ± 1.0 GBq/μmol and 10.1 ± 0.7 GBq/μmol when ^212^Pb was purified using the previous one- and novel two-column purification methods, respectively. At 80 °C, with ^212^Pb from both methods, quantitative RCYs were obtained at chelator concentrations of 10^–4^ to 10^–6^ M. At concentrations of 10^–7^ and 10^–8^ M, the RCYs with the one-column ^212^Pb decreased to 88.8 ± 5.5% and 9.8 ± 1.5%, while with the two-column ^212^Pb, the RCYs decreased to 82.6 ± 6.7% and 1.7 ± 1.5%, respectively, as shown in Fig. 7. At 80 °C, the A_m_ of [^212^Pb][Pb(TCMC)]^2+^ was 16.5 ± 1.1 GBq/μmol and 17.8 ± 1.0 GBq/μmol when using ^212^Pb purified via the previous one- and novel two-column methods, respectively.

## Discussion

In our previous work, thallium targets prepared by mechanically pressing metallic Tl into aluminum backings were only able to withstand currents up to 8 μA and ^203^Pb elutes were found to contain a stable Pb concentration greater than 400 ppb^9^. Together, these factors contributed to the low specific activity of the ^203^Pb product, which could only be overcome by modifying the target manufacturing procedure to increase thermal stability and produce more ^203^Pb at EOB. In the previous one-column purification procedure, the irradiated Tl targets were dissolved in 20 mL of 2 M HNO_3_ at 125 oC and after cooling, the target solution was loaded onto a single extraction chromatographic resin (PB resin), followed by washing with 2 M HNO_3_ (5 mL), and eluting with 1 M NH_4_OAc (pH 7, 3 mL)^9^. This method was attractive as it was rapid, simple, and also compatible with ^212^Pb purification from a ^228^Th/^212^Pb generator; the ^228^Th was purified from the Th waste of a ^232^Th proton spallation process on TRIUMF’s 500 MeV cyclotron used to produce ^225^Ac^9,24^. Although this purification process was rapid and simple, Tl and ^232^Th were present in the ^203^Pb and ^212^Pb elutes at high concentrations of 58.2 ± 35.4 ppm and 24.3 ± 16.2 ppm, respectively^9^, and thus there was a great need for the development of a new purification procedure that would reduce metal impurities in the ^203/212^Pb eluate(s).

To improve the thermal contact conductance of the thallium with the backing material, targets were manufactured via electroplating onto silver backings. Previous reports employed copper as the backing material^14^; however, given its higher thermal conductivity, silver was chosen for this work. Furthermore, the electroplating method selectively deposits Tl, which minimizes the amount of stable Pb incorporated into the deposit. This is in contrast to the pressing of Tl metal, where existing impurities are incorporated into the target, posing greater challenges during target processing and purification. Using constant current electrolysis, a maximum current density of 2.3 mA cm^−2^ was employed to minimize dendrite formation. The silver-backed electroplated targets have been successfully irradiated at currents up to 20 μA, 2.5 times greater than the previous maximum current, and will be tested with higher currents and enriched ^203^Tl targets in future studies.

Despite the capacity factor (k′) of Pb^2+^ on PB resin being nearly 100 times greater than the k′ of Tl^+^ in 2 M nitric acid^25^, with approximately 350 mg of Tl in each target, extraction chromatographic resins, including PB resin, can be overloaded beyond their capacity, which can result in variability and breakthrough. In our previous method, 320–330 mg of thallium metal was dissolved in 2 M HNO_3_ and passed through 60 mg of resin, yielding a thallium concentration of 58.2 ± 35.4 ppm in the eluate^9^. This high Tl burden resulted in variable Tl concentrations between batches, ultimately affecting the reproducibility of radiolabeling yields, particularly for chelators sensitive to competing non-radioactive metal impurities. In order to reduce the Tl concentration of the elute, we exploited differences in the solubility of TlNO_3_ at different temperatures to precipitate the majority (81.0 ± 4.5%, n = 4) of the thallium by cooling the target solution to 0 °C prior to loading on the first PB resin column. As a result, in combination with an additional 5 mL of 2 M HNO_3_ in the PB resin wash, the Tl concentration in the 8 M HCl elute was reduced to 2.2 ± 0.5 ppm (n = 3), which correlates to over a 26-fold decrease in concentration. In addition to reducing Tl burden, the precipitated TlNO_3_ can be collected, and the Tl recycled, which is of great importance when using costly enriched materials. To further improve recycling yield, the load and wash of the PB resin can be evaporated which results in a Tl recovery of 94.8 ± 1.0% (n = 4). The recovered Tl was confirmed to be in the + 1 oxidation state as TlNO_3_ by using a colorimetric method^23^. The recovered TlNO_3_ was successfully electroplated, with the resulting targets showing no statistical difference in final target mass, or ^203^Pb produced at EOB, compared to non-recycled TlNO_3_ or Tl_2_SO_4_.

Ideal radiochemical separations involve the use of a minimal number of columns and no evaporation which would complicate automation and minimize potential losses. Additionally, it can be most beneficial when testing a wide range of chelators for the activity to be eluted in an easily neutralized medium, so that optimization can be performed for each chelator. For the first (PB) column, it was still most ideal to use a nitric acid medium to separate the ^203^Pb or ^212^Pb from Tl and Th, respectively, as Pb^2+^ has high k^′^_Pb_ at all nitric acid concentrations. Initially studies utilized an eluate composed of 0.01 M HNO_3_^25^. However, this resulted in a large elution volume and thus was not further investigated. 8 M hydrochloric acid can be an effective desorption agent from PB resin as the k^′^_Pb_ is less than 10 at higher acid concentrations^25^. This resulted in 94.8 ± 2.7% and 94.9 ± 0.8% (n = 4) of the ^203^Pb and ^212^Pb to be eluted from this resin in 2 mL of 8 M HCl, respectively. However, 8 M HCl is not easily compatible with radiolabeling and there were still high concentrations of Tl (2.2 ± 0.5 ppm, ^203^Pb), Ag (3.3 ± 0.3 ppm, ^203^Pb), and Th (37.7 ± 3.2 ppm, ^212^Pb) present, and as such, the use of a second column to improve chemical purity and reduce acid concentration was required.

To eliminate the need for acid exchange (e.g. HNO_3_) and evaporation of the 8 M HCl eluate before loading the second column, we searched for distribution coefficients (D) of Pb^2+^ with HCl on strong base type I anion exchange resins^26–28^. Through method development studies (see Supporting Information), Dowex-1X8 resin was chosen as the resin for the second column. The adsorption of Pb^2+^ is low in dilute HCl (D = 1 in 0.05 M HCl), reaches a maximum at 1.5 M HCl (D = 25), and decreases such that there is minimal adsorption (D < 1) at concentrations greater than 8 M HCl^26–28^. As a result, ^203/212^Pb can be loaded and washed in 1–2 M HCl and eluted in 0.01 M HCl. Additionally, there is little to no adsorption of Th^4+^ and Tl^+^ under these conditions and a higher adsorption of Ag^+^ over Pb^2+^, which should result in decreases of these main contaminants^28^. Despite the difference in D at varying HCl concentrations, these values are still relatively low and thus it was critical to optimize the loading and wash volumes to reduce the breakthrough of ^203^Pb and ^212^Pb. As a result, only the first 1 mL of the 8 M HCl elute, which contained 90.0 ± 2.4% and 89.0 ± 1.6% of the initial ^203^Pb and ^212^Pb, respectively, was taken and diluted to 2 M HCl rather than to 1.25 M, at which the D is the highest, to minimize the loading volume while still maintaining sufficient adsorption to the resin. The resin was then washed in 1 M HCl to further remove metal impurities and reduce the acid concentration in the final elute as the ^203^Pb/^212^Pb is eluted with 0.01 M HCl to allow for easier neutralization by radiolabeling buffers. The flow rate was also found to be critical to the successful use of the Dowex-1X8 resin, as high flow rates (> 0.5 mL/min) would cause activity breakthrough. To minimize losses, the column was loaded and washed by gravity. Despite this limitation, the low volume of the load and wash allows for the entire purification procedure, for either ^203^Pb or ^212^Pb, to be completed in 2 h.

The most important benefit of this novel method over the previous one-column method is the reduction in the mass and concentrations of Tl and Th contaminants in the ^203^Pb and ^212^Pb elutes, respectively. With the previous method, the Tl separation factor was 1.86 × 10^3^, whereas with the novel method, a separation efficiency of 6.68 × 10^6^ was achieved, leading to a Tl:^203^Pb ratio of 122:1. Therefore, compared to the previous method, the optimized method reduced the Tl mass content 3.59 × 10^3^-fold and the Tl concentration by 2.24 × 10^3^-fold. To the best of our knowledge, this is the lowest Tl concentration in ^203^Pb reported in the literature^9,16,17^. In the blood, Tl levels above 200 μg/L are considered toxic; assuming an average of 5 L of blood in the human body, with a mass of 52 ng of thallium in the entire ^203^Pb elute, which is expected to be used only in part for an individual dose, the projected Tl injected is several orders of magnitude below toxic levels. Additionally, given the selectivity of the electroplating procedure towards Tl, reduced stable Pb concentrations of 34 ± 6 ppb were detected in the ^203^Pb elute, compared to 495 ± 218 ppb from our previously reported method. For a two hour irradiation of a natural Tl target at maximum current (8 μA^9^ vs 20 μA), the ^203^Pb specific activity increased from 18.3 ± 8.6 MBq/μg^9^ to 969.1 ± 173.9 MBq/μg (n = 3) for the previous one- and novel two-column method, respectively. This resulted in an average stable Pb:^203^Pb ratio of 6:1 which can be further reduced as (i) irradiation currents increase, (ii) enriched targets are employed, and (iii) the ^205^Tl (p, 3n) ^203^Pb reaction is utilized at higher proton energies.

For ^212^Pb, the greatest benefit of the novel two-column method over the previously employed one-column method was a 126-fold greater reduction in Th content. Previously with one column, the Th separation factor was 1.10 × 10^5^_,_ which resulted in a final elute concentration of 24.3 ± 16.2 ppm (n = 3)^9^. With the two-column method, a separation factor of 1.37 × 10^7^ was achieved to give a final Th^4+^ concentration of 291 ± 56 ppb (n = 3), which is nearly an 84-fold decrease in ^232^Th concentration in the final elute. Concentrations of previous contaminants Mg, Al, Co, and Ti, decreased even further to 15 ± 7 ppb, 2 ± 3 ppb, 0.3 ± 0.2 ppb, and 0 ppb (N.S.), respectively, thus improving the chemical purity of the ^212^Pb product. The stable Pb^2+^ concentration did not significantly differ. This has led to a ^232^Th:^212^Pb ratio of 1203:1 in the Dowex elute compared to the initial ratio of 1.65 × 10^10^:1 in the generator stock solution. This reduction in ^232^Th in the elute may explain the dramatic improvement in the ^212^Pb RCYs of Crypt-OH.

The significance and impact of the optimizations achieved through the novel method are demonstrated by the corresponding improvements in the RCYs of Pb^2+^ chelators TCMC and Crypt-OH. With a lower Tl and stable Pb content, the A_m_ of [^203^Pb][Pb(TCMC)]^2+^ and [^203^Pb][Pb(Crypt-OH)]^2+^ increased by a factor of seventeen and nine at ambient temperature, respectively, showing that improvements in ^203^Pb specific activity and chemical purity translated directly into improved RCYs. For ^212^Pb, there were no substantial changes to the RCYs of TCMC at room temperature or at 80 °C. This is most likely because the stable Pb concentration did not significantly change and TCMC is more selective towards Pb^2+^and not affected drastically by Th^4+^. With the previous one-column method, the average Th concentration was 24.3 ± 16.2 ppm, which led to a ^232^Th:^212^Pb of 1.5 × 10^5^:1, which decreased to 1203:1 with the novel, two-column method. Interestingly, in the case of Crypt-OH, no radiolabeling was observed with ^212^Pb from either method at room temperature, despite the successful quantitative radiolabeling with ^203^Pb. This suggests that the cryptand is highly sensitive to the concentration of Th^4+^. However, moderate radiolabeling yields were observed at 80 °C with the novel, two-column method, suggesting that the ^212^Pb radiolabeling may be kinetically driven, but this is beyond the scope of this paper and will be a focus of later studies. With a 126-fold decrease in the ^232^Th:^212^Pb ratio, a 129-fold improvement in the A_m_ of [^212^Pb][Pb(Crypt-OH)]^2+^ was observed at 80 °C, demonstrating the importance of the chemical purity of ^212^Pb for this chelator.

## Conclusions

We have developed a target manufacturing method that involves electroplating thallium onto silver. This resulted in a target with high thermal stability, one capable of withstanding higher irradiation currents, and leading to a significant increase in ^203^Pb production when irradiated with a 13 MeV cyclotron. These improvements to the target manufacturing procedure, due to higher allowable current and lower stable Pb content, have resulted in a nearly 53-fold increase in the specific activity of ^203^Pb. Additionally, we have developed a novel purification procedure that, to the best of our knowledge, has produced ^203^Pb/^212^Pb of the highest chemical purity reported to date from an academic laboratory. The specific activity and ^203^Pb: stable Pb ratio are expected to improve in future studies as enriched targets are manufactured and irradiated with high proton energies and currents towards a maximum of 26.5 MeV; this will allow production of ^203^Pb at a level suitable for clinical use. Further, as ^232^Th irradiations are conducted at higher currents and for longer durations, this purification procedure will allow researchers to easily produce high specific activity ^212^Pb for pre-clinical purposes to meet the growing demand and interest in this theranostic pair that is seeing increasing clinical use.

## Supplementary Information

Supplementary Information.

## Data Availability

The datasets generated and analyzed during the current study are available from the corresponding author on reasonable request.
