# Supplementary material for: Optimized production, purification, and radiolabeling of the 203Pb/212Pb theranostic pair for nuclear medicine
Source: Sci Rep. 2023 Jun 30;13:10623. doi: 10.1038/s41598-023-37313-8 (PMC10313663; doi:10.1038/s41598-023-37313-8)
Supplement: Supplementary file 1 — Supplementary Information. [file 41598_2023_37313_MOESM1_ESM.pdf]

Supporting Information

Optimized Production, Purification, and Radiolabeling of the $^{203}Pb/^{212}Pb$ Theranostic Pair for Nuclear Medicine

Brooke L. McNeil[1,2], Simona A. Mastroianni[1], Scott W. McNeil[1], Stefan Zeisler[3], Joel Kumlin[3], Sogol Borjian[3], Anthony W. McDonagh[2], Paul Schaffer[1,2,3,4], Caterina F. Ramogida[1,2]*

[1]Life Sciences Division, TRIUMF, Vancouver BC, V6T 2A3, Canada.
[2]Department of Chemistry, Simon Fraser University, Burnaby BC, V5A 1S6, Canada.
[3]ARTMS Inc., Burnaby, BC V5A 4N5, Canada.
[4]Department of Radiology, University of British Columbia, 2775 Laurel St, Vancouver, BC V5Z 1M9, Canada.
*corresponding email: caterina_ramogida@sfu.ca

Gamma-ray spectra

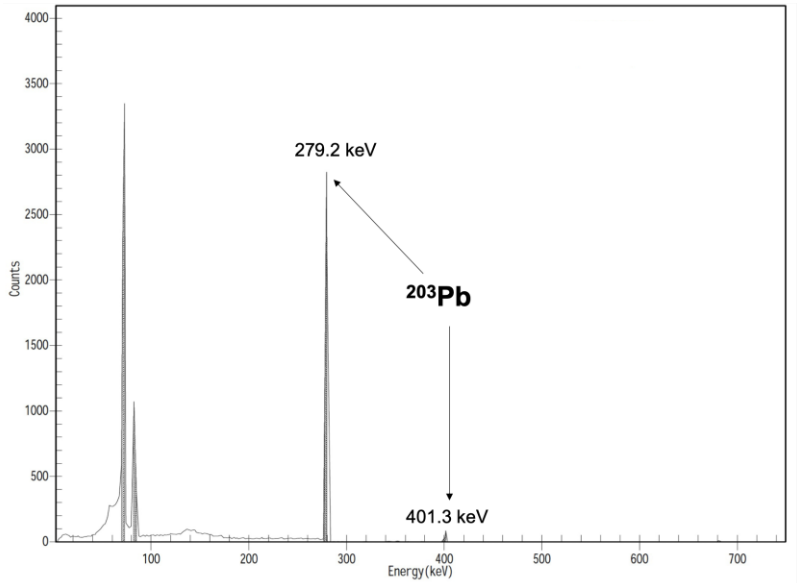

Figure S1. Gamma-ray spectrum of the ^{203}Pb Dowex-1x8 elute.

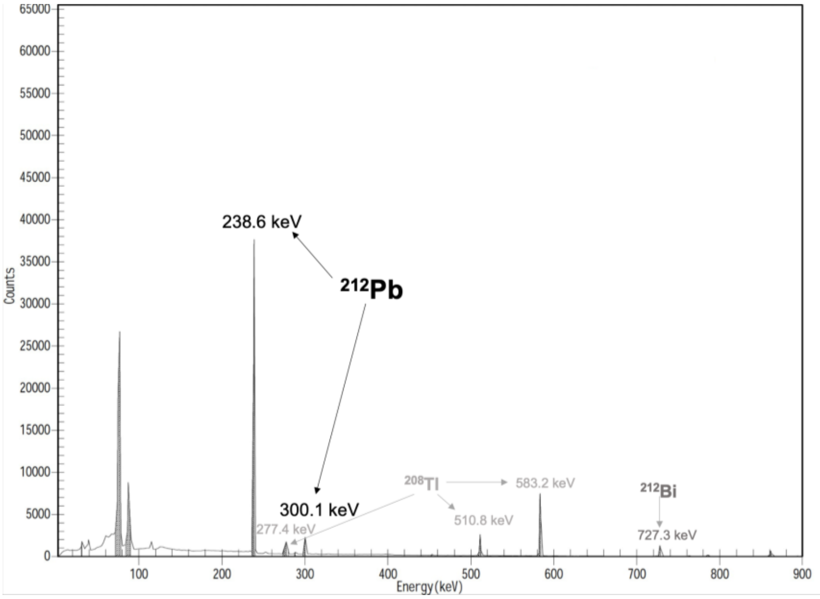

Figure S2. Gamma-ray spectrum of the ^{212}Pb Dowex-1x8 elute.

Dowex-1X8 Resin - Method Development

When anion exchange resins were being considered for use as a second column in our two-column purification procedure, we evaluated both Dowex-1X8 (200-400 mesh, Cl form, Pittsburgh, PA) and AG-1X8 (50-100 mesh, Cl form, Biorad, Mississauga, ON) resins. AG-1X8 and Dowex-1X8 resins share the same structure (type I, strong base anion exchangers with quaternary amine functional groups on styrene divinyl benzene polymeric beads), but AG-1X8 is an analytical grade resin, whereas Dowex-1X8 is not. Analytical grade resins typically contain less metal contaminants and have a more narrow particle size distribution that can lead to superior resolution, which could result in smaller elute volumes, ideal for radiochemical separation. However, this resin is nearly twice the price of Dowex-1X8.

In order to determine which resin was more useful for our applications, and what column volume should be used for a loading volume of at least 4 mL, 100 mg of each resin (column volume of ~100 μL) was first used. The resin was packed as a slurry in deionized water into a 1 mL polypropylene cartridge and preconditioned with 5 mL of deionized water and 5 mL of 2 M HCl. The loading solution containing the diluted Pb™ resin elute (in 2 M HCl) was loaded onto the column by gravity before eluting with 0.01 M HCl at 1.5 mL/min. Gamma spectroscopy was used to quantify the ^{203}Pb in the load and elute fractions as at this point in the investigative process, the 1 M HCl wash was not yet employed, as shown in **Figure S3**.

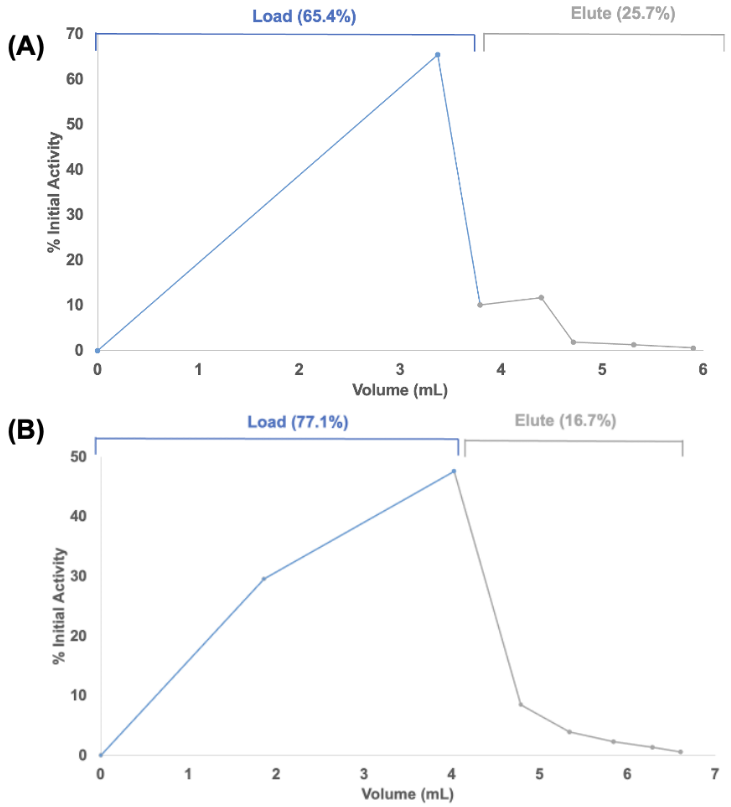

Figure S3. Average elution profile of ^{203}Pb (~4 mL in 2 M HCl) purification on (A) 100 mg of Dowex-1X8 resin and (B) 100 mg of AG-1X8 resin (n=1).

Despite this only being one replicate, it was clear that this column volume was too small and thus had to be increased. For the next set of experiments, 500 mg of resin (column volume ~530 μL) was packed as a slurry into a 1 mL polypropylene cartridge and preconditioned with 10 mL of deionized water and 10 mL of 2 M HCl. The columns were loaded by gravity and eluted with 0.01 M HCl at 1.5 mL/min, with the elution profiles shown in **Figure S4**.

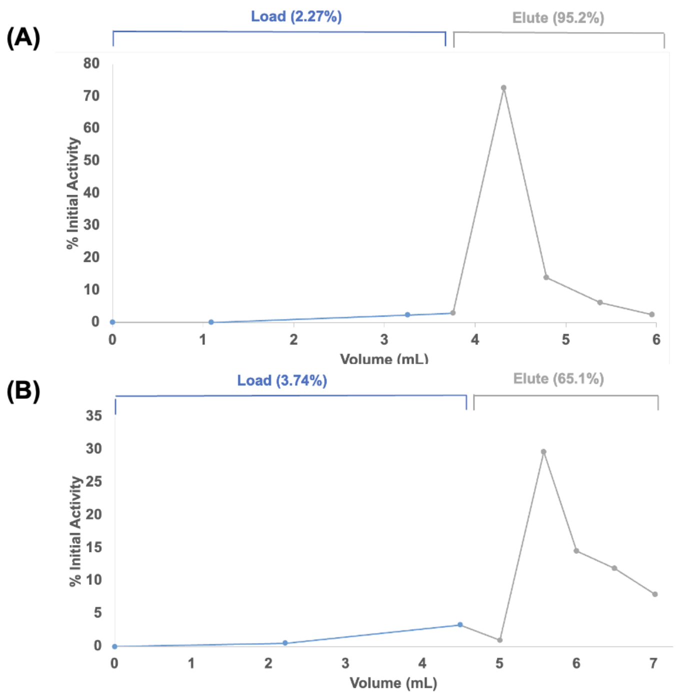

Figure S4. Average elution profile of ^{203}Pb (~4 mL in 2 M HCl) purification on (A) 500 mg of Dowex-1X8 resin and (B) 500 mg of AG-1X8 resin (n=1).

Initially, it was expected that the elution profiles would be opposite as AG-1X8 should offer greater resolution. However, due to the drastic, unexpected difference in the elution profiles in **Figure S4**, and the lower cost, it was decided to proceed with Dowex-1X8 as the resin of choice. To further determine if the ^{203}Pb lost in the load could be further reduced, 1000 mg of Dowex-1X8 resin was prepared as previously described and identical loading and elution conditions were used.

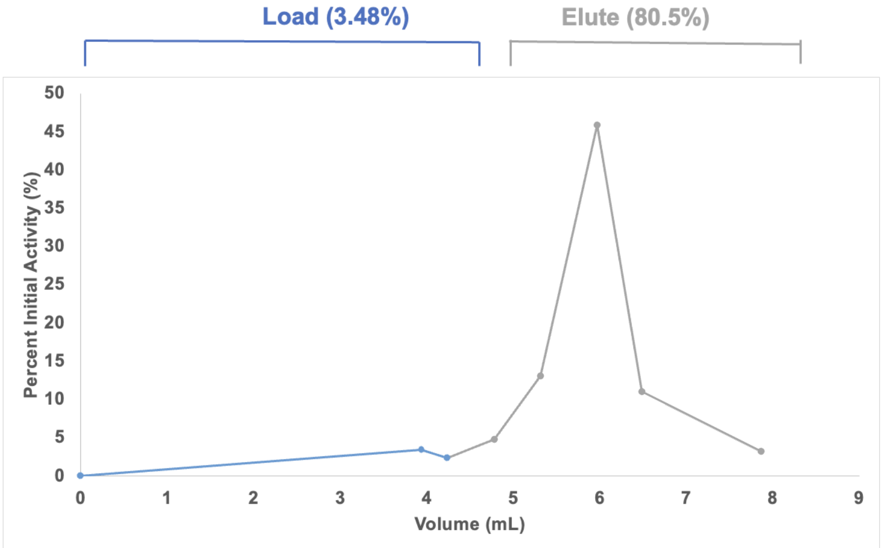

Figure S5. Average elution profile of ^{203}Pb (~4 mL in 2 M HCl) purification on 1000 mg of Dowex-1X8 resin. (n=1)

With a 1000 mg column, the ^{203}Pb in the load did not decrease and the elution volume and profile began to broaden as more 0.01 M HCl was required to elute the ^{203}Pb from the larger column, as expected. Since it is desirable for the elute volume to be as low as possible, 500 mg was the finalized column volume used in this novel purification procedure.

Representative iTLCs

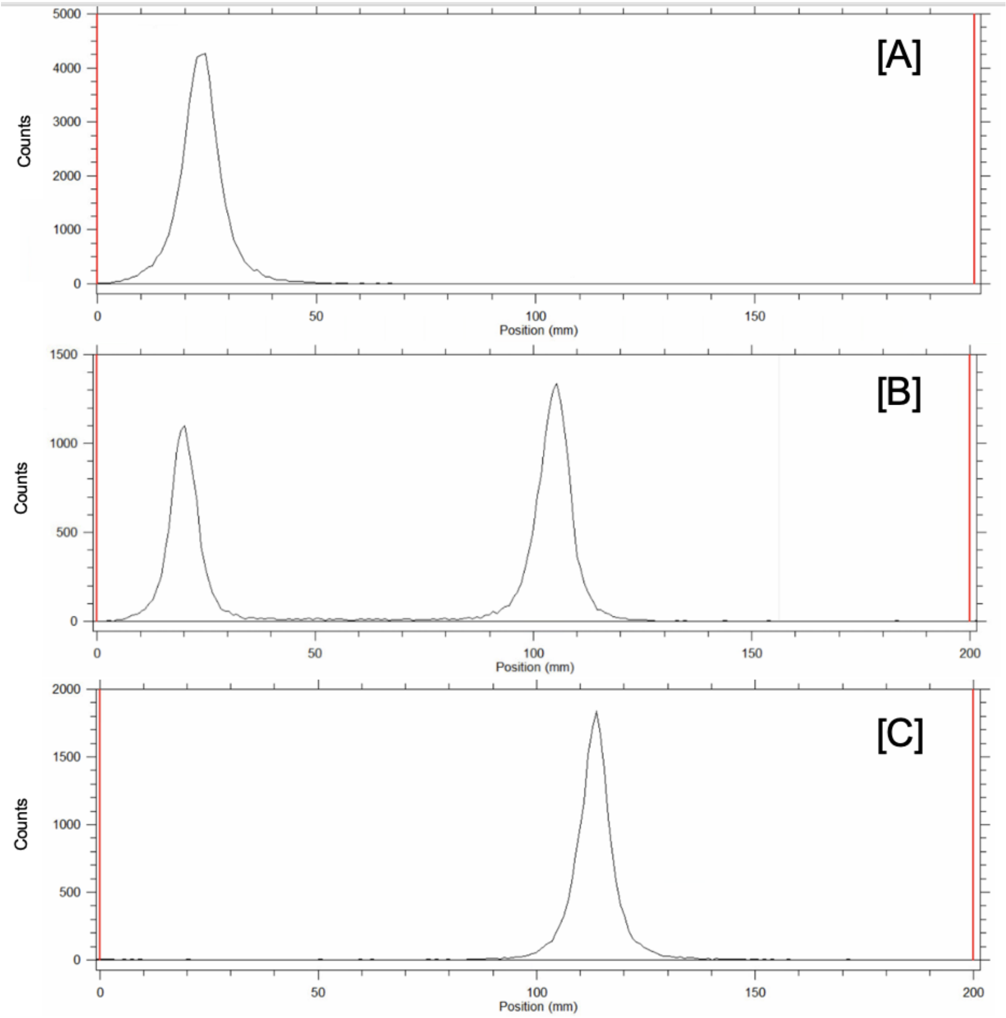

Figure S6. Representative iTLC radio-chromatograms for ^{212}Pb radiolabelling. [A] 10^{-6} M TCMC labelled with 100 kBq ^{212}Pb at room temperature (0.1 M NH_4OAc, pH 7, 1 h), [B] 10^{-7} M TCMC labelled with 100 kBq ^{212}Pb at room temperature (0.1 M NH_4OAc, pH 7, 1 h), and [C] unlabelled ^{203}Pb (negative control) 1 h aliquot spotted onto SA iTLC plates, developed using EDTA (50 mM, pH 5.0) as the mobile phase.

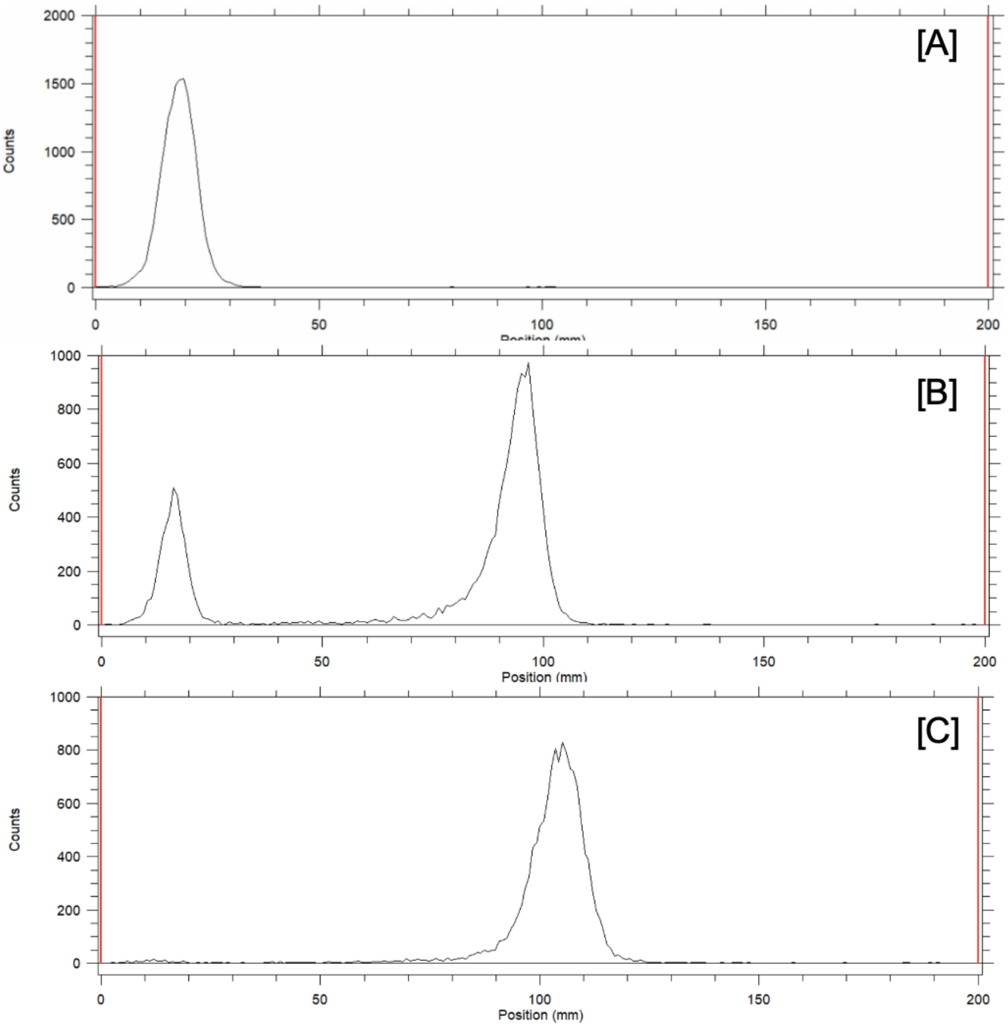

Figure S7. Representative iTLC radio-chromatograms for ^{203}Pb radiolabelling. [A] 10^{-6} M TCMC labelled with 85 kBq ^{203}Pb at room temperature (0.1 M NH_4OAc, pH 7, 1 h), [B] 10^{-8} M TCMC labelled with 85 kBq ^{203}Pb at room temperature (0.1 M NH_4OAc, pH 7, 1 h), and [C] unlabelled ^{203}Pb (negative control) 1 h aliquot spotted onto SA iTLC plates, developed using EDTA (50 mM, pH 5.0) as the mobile phase.

^{212}Pb Radiolabeling

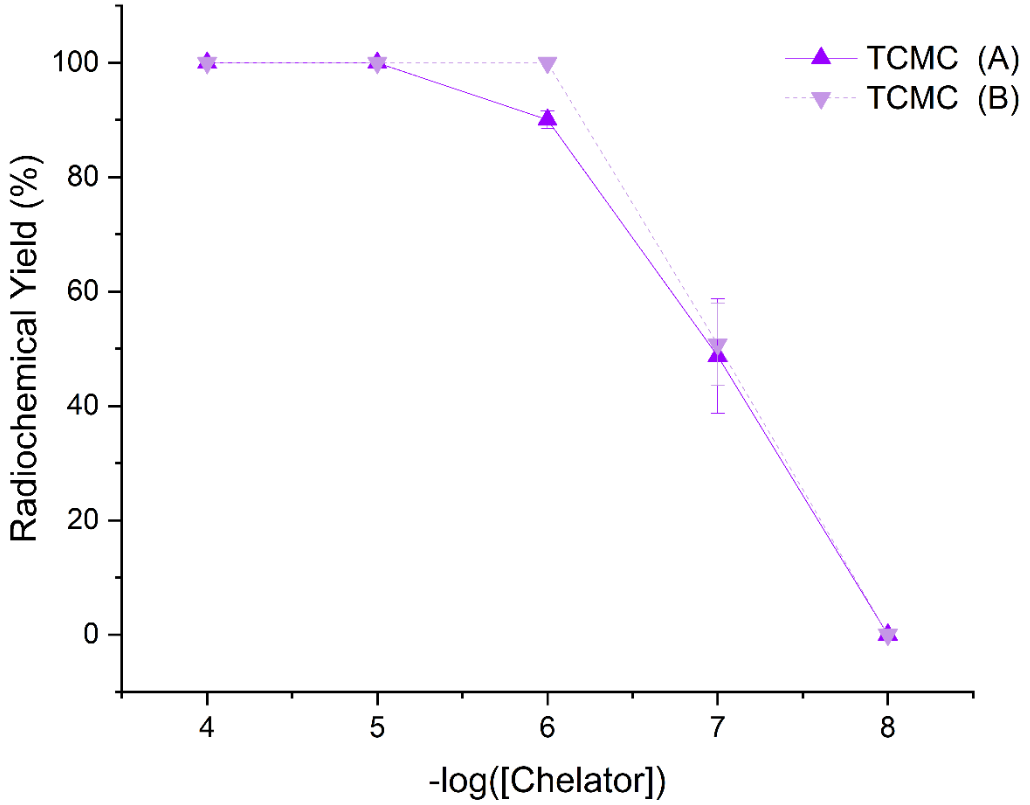

Figure S8. Radiochemical yields (%) for ^{212}Pb (100 kBq) labeling reactions at pH 7 (0.1 M NH_4OAc), room tempeature, and 1 h at chelator concentrations of 10^{-4} to 10^{-8} M using ^{212}Pb produced via the one-column[9] (A) and two-column (B) method. (n = 3)

<u>Sample Calculations</u>

Specific activity

Irradiation parameters = 2 hour irradiation of a natural Tl target at maximum current (8 μA[1] vs 20 μA)
Activity produced at EOB = 27.3 ± 4.7 MBq[1] and 65.9 ± 2.3 MBq
Average elute mass of Pb (n = 3) = 1.485 ± 0.654 μg for previous method[1] and 68 ± 12 ng (6.8×10^{-2} μg)

Previous method specific activity = $\frac{27.3 \pm 4.7 \text{ MBq}}{1.485 \pm 0.654 \text{ μg}}$ = 18.38 ± 8.6 MBq/μg[1]

Specific activity in this study = $\frac{65.9 \pm 2.3 \text{ MBq}}{6.8x10^{-2} \pm 1.2\text{x}10^{-2} \text{ μg}}$ = 969.1 ± 173.9 MBq/μg

Apparent molar activity (AMA)

Reaction volume = 100 μL (1.0×10^{-4} L)
Concentration of lowest radiolabeling >50% = 1.0×10^{-7} M
Number of moles in a 100 μL reaction at a concentration of 1.0×10^{-7} M = 1.0×10^{-11} moles (1.0×10^{-5} μmoles)

Activity used in reaction = $8.5x10^{-2}$ MBq (85 kBq)
MBq/μmol in reaction = 8500 MBq/μmol
Account for only 86.7 ± 2.4% being incorporated at 10^{-7} M = 8500 MBq/μmol x 0.8877
AMA = 7369.5 MBq/μmol

Photograph of Average Electroplated Tl Target

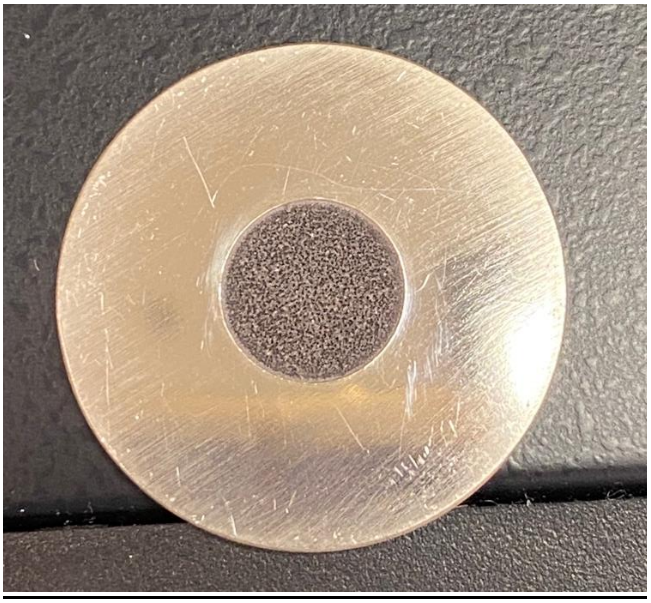

Figure S9. Photograph of a representative silver-backed, electroplated ^{nat}Tl target.

References

(1) McNeil, B. L.; Robertson, A. K. H.; Fu, W.; Yang, H.; Hoehr, C.; Ramogida, C. F.; Schaffer, P. Production, Purification, and Radiolabeling of the 203Pb/212Pb Theranostic Pair. *EJNMMI Radiopharm Chem* **2021**, *6* (6).
